# Parameter Scaling for Epidemic Size in a Spatial Epidemic Model with Mobile Individuals

**DOI:** 10.1371/journal.pone.0168127

**Published:** 2016-12-14

**Authors:** Chiyori T. Urabe, Gouhei Tanaka, Kazuyuki Aihara, Masayasu Mimura

**Affiliations:** 1 Institute of Industrial Science, The University of Tokyo, Tokyo, Japan; 2 Graduate School of Engineering, The University of Tokyo, Tokyo, Japan; 3 Meiji Institute for Advanced Study of Mathematical Sciences, Meiji University, Tokyo, Japan; Universidad Nacional de Mar del Plata, ARGENTINA

## Abstract

In recent years, serious infectious diseases tend to transcend national borders and widely spread in a global scale. The incidence and prevalence of epidemics are highly influenced not only by pathogen-dependent disease characteristics such as the force of infection, the latent period, and the infectious period, but also by human mobility and contact patterns. However, the effect of heterogeneous mobility of individuals on epidemic outcomes is not fully understood. Here, we aim to elucidate how spatial mobility of individuals contributes to the final epidemic size in a spatial susceptible-exposed-infectious-recovered (SEIR) model with mobile individuals in a square lattice. After illustrating the interplay between the mobility parameters and the other parameters on the spatial epidemic spreading, we propose an index as a function of system parameters, which largely governs the final epidemic size. The main contribution of this study is to show that the proposed index is useful for estimating how parameter scaling affects the final epidemic size. To demonstrate the effectiveness of the proposed index, we show that there is a positive correlation between the proposed index computed with the real data of human airline travels and the actual number of positive incident cases of influenza B in the entire world, implying that the growing incidence of influenza B is attributed to increased human mobility.

## Introduction

Pandemics are recognized as a serious threat and concern all over the world. To cope with this issue, considerable efforts have been made for investigating the mechanism of the spread of infectious diseases and finding possible control measures for preventing epidemic outbreaks. In particular, recent epidemics are more likely to spread in a broad area than before, because of the effect of worldwide human mobility through a variety of transportation networks [1, 2]. Mathematical models have been a powerful tool to reveal the effect of human behaviour on epidemic spreading and examine the effectiveness of countermeasures against emerging and re-emerging infectious diseases. To study spatial spreading of epidemics which cannot be treated by deterministic compartmental models assuming a well-mixed population [3, 4], many mathematical models have incorporated both spatial structures and host mobility. The two major classes of such computational models are the individual-based (agent-based) models and the metapopulation models [5].

The individual-based models have been widely used to simulate the spreading of epidemics at a microscopic level (i.e. a level of individual hosts), assuming stochastic processes for infection, recovery, birth, death, and movement events [5]. Many recent models have incorporated directly or indirectly the real data of population distributions, human mobility patterns, and human proximity, for investigating the spatiotemporal dynamics of epidemic spreading and assessing the effects of preventive measures [2, 6–11]. The advantage of individual-based models is that they can enhance the reality of simulations by appropriately setting many parameters estimated from available data sources. However, the outcome of the simulation can be largely variable from trial to trial due to the repeated stochastic calculations and highly dependent on the parameter conditions as well as initial conditions. Therefore, it is tough to gain an insight into essential factors that mainly contribute to the spatial spreading of epidemics in individual-based models without a large number of simulations.

On the other hand, metapopulation models describe the epidemic dynamics of subpopulations in spatially separated patches connected via migration pathways and deal with human mobility patterns at a macroscopic level [12, 13]. The statistical properties of various human traveling patterns via transportation networks, such as airlines, railways, and commuting roads, have been well incorporated into the metapopulation models [14–25]. Compared with individual-based models, the metapopulation models are analytically tractable. The previous studies have theoretically derived the global invasion threshold [18, 20, 21] and the critical intervention threshold for containment of epidemics [26] in metapopulation models with heterogeneous patch connectivity. However, the stochastic nature of the movement of individual hosts is not explicitly considered in the metapopulation models because they only describe the mobility dynamics of subpopulations.

In various types of epidemic models, it has been the central issue how the final epidemic size is determined by the individual system parameters or the composite of them. The important measures which determine the epidemic threshold and predict the final epidemic size have been derived for several model types, e.g. the basic reproduction number for the standard compartment models [4, 5] and the global invasion threshold for the metapopulation models [20, 21]. However, such a measure is yet to be established for individual-based models with stochastic mobility of individuals. This issue has motivated the present study.

In this study, we aim to perceive the key factors that govern the final epidemic size in spatial epidemic models with heterogeneous mobility of individuals and clarify how each key factor contributes to the final epidemic size. For this purpose, we employ a susceptible-exposed-infectious-recovered (SEIR) model with mobile individuals in a square lattice. Since multiple individuals can be located at a single lattice site, our model is similar to a spatial metapopulation model where each site of the lattice corresponds to the patch containing a subpopulation [5, 16, 27]. However, in our model, the subpopulation dynamics is given not by deterministic differential equations but by individual-based stochastic processes. Spatial metapopulation models have been frequently used for data-driven simulations to investigate geographical propagation of measles [28–30], influenza [31, 32], and smallpox [10, 19], but universal properties of such models have been less studied so far. The purpose of our study is to clarify how the final epidemic size is determined by the interplay between disease characteristics and spatial mobility of individuals. Note that the time-varying contact patterns in our model cannot be represented by the lattice-based cellular automata models where contact relationships are static [33–39].

We separate the infected individuals into the exposed ones and infectious ones. The exposed individuals are not able to transmit a disease to other susceptible individuals, whereas the infectious individuals are able to do. We assume that the mobility of infectious individuals can be lower than that of the other classes of individuals due to severe symptoms and/or travel restrictions [40–42]. Under the individual’s mobility depending on its internal state, we first perform individual-based simulations of the SEIR model in a square lattice and clarify the interplay between the latent period and the mobility for the final epidemic size. We find that the distance that each infected individual moves during the latent and infectious periods plays a decisive role for the final epidemic size. Then, based on the theory of diffusion processes, we present an index giving an epidemic threshold that is almost invariant under parameter scaling. Finally, we demonstrate a strong correlation between the proposed index incorporating the real data of human airline travels and the incidence of influenza B. Our result implies an important role of human mobility on the final epidemic size.

## Methods

### Models

**Fig 1:**
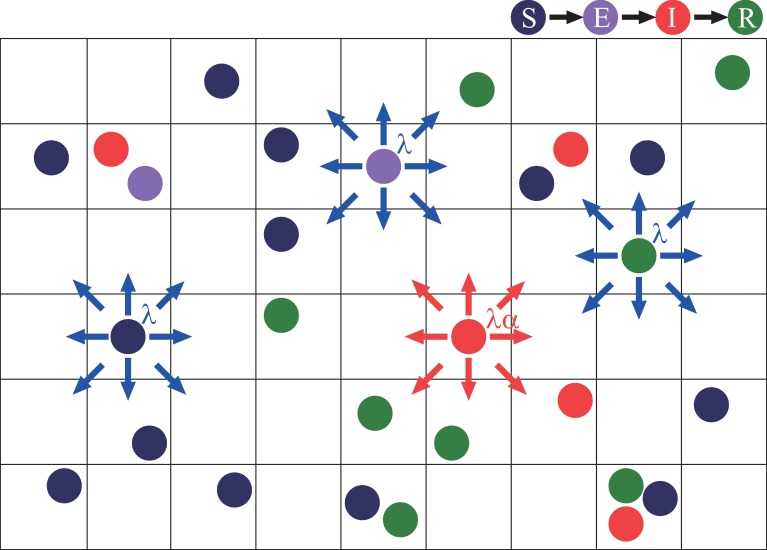
Schematic illustration of the spatial SEIR model with mobile individuals in the square lattice. The individuals randomly hops from site to site. Each of the susceptible (S), exposed (E), and recovered (R) individuals hops to one of the eight neighbouring sites with the hopping rate λ, while each of the infectious individuals (I) hops similarly with the rate ***λα*** where **1−*α*** represents the mobility reduction rate.

We adopt the spatial SEIR model in which individuals move randomly on a two-dimensional lattice with the periodic boundary condition as illustrated in Fig 1. As time goes by, each individual can change its internal state as well as its spatial position.

First, we explain the state transition of individuals. Each individual is in a susceptible state (S), an exposed state (E), an infectious state (I), or a recovered state (R). Note that both exposed and infectious individuals are infected but only infectious ones are capable of transmitting the disease to susceptible ones. The individuals sharing the same lattice site are regarded to be in contact with each other. Susceptible individuals can be infected only when they share a lattice site with one or more infectious individuals. We assume that a susceptible individual becomes an exposed one with probability *p* for a contact with each infectious individual in one unit of time. If a susceptible individual shares a site with *ν* infectious individuals, then the susceptible individual becomes an exposed one with probability 1 − (1 − *p*)^*ν*^ in one unit of time. This probability is independent of the numbers of susceptible, exposed, and recovered individuals positioned at the site. An exposed individual becomes an infectious individual after a latent period of fixed length *τ*_E_. Then, after an infectious period of fixed length *τ*_I_, an infectious individual becomes a recovered individual who never again becomes susceptible.

Second, we describe the mobility of individuals. Each individual is positioned at one lattice site, and then, in one unit of time, will hop from site to site in a probabilistic manner. Multiple individuals can be positioned at a single site. It is possible that a site contains no individuals. With a single hop, an individual can move to one of its eight neighbouring sites as indicated in Fig 1. The probability that susceptible, exposed, and recovered individuals hop to each one of the destination sites is given by the normal hopping rate *λ* with $0<\lambda \leq \frac{1}{8}$, while infectious individuals can have a lower hopping rate *αλ* with 0 ≤ *α* ≤ 1 because they are usually less active and sometimes the target of social distancing [40–42]. The value 1 − *α* represents the mobility reduction rate of infectious individuals. Both the normal and lower hopping rates are independent of the situation of the destination site. In the case of *α* = 0, infectious individuals do not move, while in the case of *α* = 1 there is no mobility reduction.

Finally, the simulation methods are described. The number of susceptible, exposed, infectious, and recovered individuals at time *t* are denoted by *n*_S_(*t*), *n*_E_(*t*), *n*_I_(*t*), and *n*_R_(*t*), respectively. Since birth and death of individuals are neglected in our model, the total number of individuals, given by *n+*1 *≡ n*_S_(*t*) *+ n*_E_(*t*) *+ n*_I_(*t*) *+ n*_R_(*t*), remains constant with time. At the initial condition, all the individuals are distributed randomly in the lattice sites. They are all susceptible except for a single infectious individual which is randomly chosen. Therefore, we have *n*_S_(0) *= n*, *n*_I_(0) = 1, and *n*_E_(0) *= n*_R_(0) = 0. The initial density of susceptible individuals is denoted by $\rho_{0}\equiv \frac{n}{L^{2}}$ for an *L* × *L* square lattice. At the end of an epidemic outbreak, it follows that *n*_E_(*t*) *= n*_I_(*t*) *=* 0 and *n*_S_(*t*) + *n*_R_(*t*) = *n+*1. We define the proportion of the recovered individuals as *r*(*t*) ≡ *n*_R_(*t*)*/*(*n+*1). Then *r*_∞_ ≡ lim_*t*→∞_*r*(*t*) represents the final size of an epidemic. The final size *r*_∞_ depends on the parameters, *ρ*_0_,*τ*_E_, *τ*_I_,*p*,*λ*, and *α*. The parameter values are set at *ρ*_0_ = 0.4 (*L* = 500, *n* = 10^5^) and *p* = 1, unless otherwise noted.

## Results

### Interplay between the latency period and the mobility

**Fig 2:**
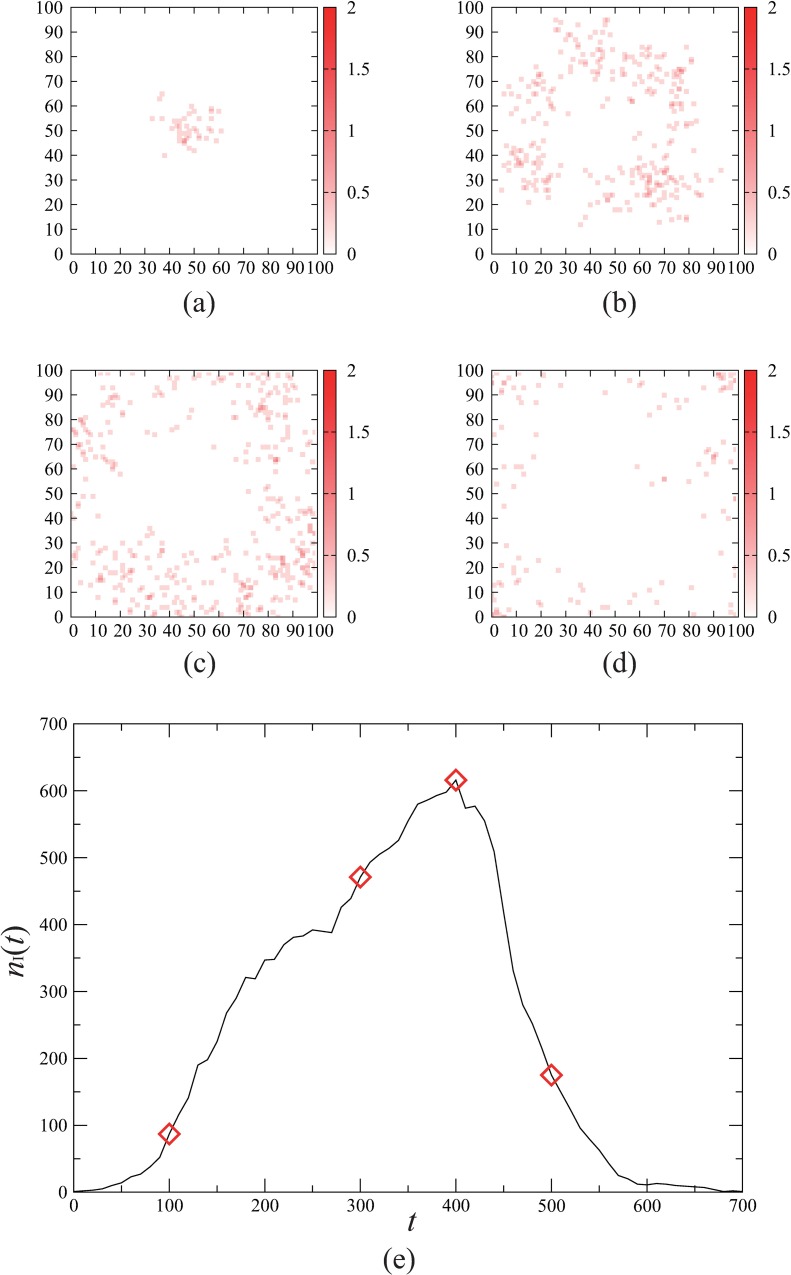
Spatial spreading of an epidemic. Time evolution of the epidemic spreading in individual-based simulations of the spatial SEIR model is shown. Initially all the individuals are susceptible except for a single infectious individual located at the centre of the lattice space. The parameter values are set at *L* = 100, *n* = 10^4^, *τ*_E_ = 8, *τ*_I_ = 16, *p* = 1.0, $\lambda =\frac{1}{8}$, and α = 0.5. (a)-(d) The snap shots of the spatial distribution of the infectious individuals for (a) *t* = 100, (b) *t* = 300, (c) *t* = 400, and (d) *t* = 500. The density of the infectious individuals in each site is indicated by the colour strength. (e) The time course of the number *n*_I_(*t*) of infectious individuals. The diamonds correspond to the patterns (a)-(d).

Fig 2 demonstrates the spatial spreading of an epidemic in our model with lattice size *L =* 100. The snap shots in Fig 2(A)–2(D) represent the time evolution of the spatial distributions of the infectious individuals. We see that an infectious individual initially located close to the centre causes the diffusion of the infection towards the lattice boundary. This spatial pattern is pretty irregular due to the stochastic mobility of the individuals compared with the ring-shaped propagation generated by other lattice-based models [5, 39]. The number of the infectious individuals, *n*_I_(*t*) varies with time as shown in Fig 2(E). Initially *n*_I_(*t*) grows rapidly until the highest peak is achieved, and then, almost monotonically decays to zero. We can evaluate the final size *r*_∞_ of this outbreak by the value of *r*(*t*) at the end of the outbreak. Whether the initial infectious individual brings about a global spread of infection or not depends on the parameter conditions as well as initial conditions.

**Fig 3:**
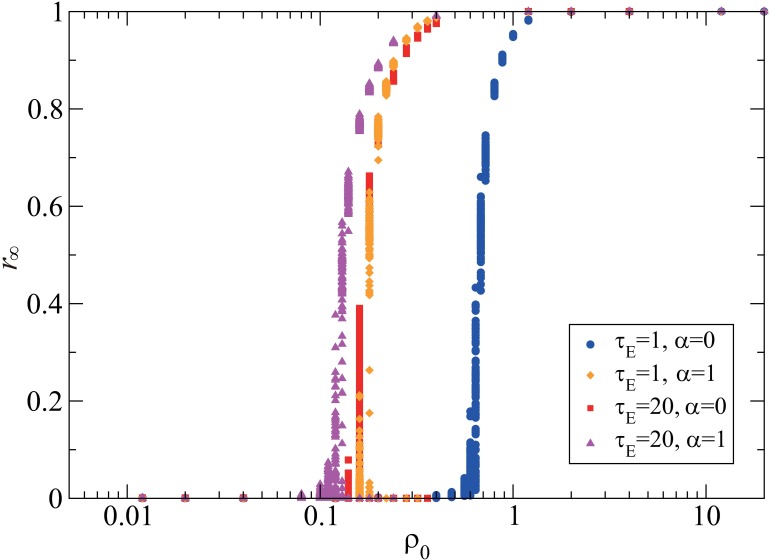
Interplay between the latent period and the mobility for the final size. The final size ***r***_∞_ is plotted against the population density ***ρ***_**0**_. For each parameter value, the results of 100 simulations are plotted. The four cases are compared with respect to the latent period and the mobility, including ***τ***_**E**_ = **1** and ***α*** = **0** (blue circles), ***τ***_**E**_ = **1** and ***α*** = **1** (orange diamonds), ***τ***_**E**_ = **20** and ***α*** = **0** (red squares), and ***τ***_**E**_ = **20** and ***α*** = **1** (purple triangles). The other parameter values are set at ***L*** = **500**, ***τ***_**I**_ = **20**, ***p*** = **1**, and $\lambda =\frac{1}{9}$.

The population density *ρ*_0_ is one of the essential parameters that give a critical epidemic threshold separating the non-epidemic and epidemic regimes [20]. The larger the epidemic threshold is, the less likely to spread the epidemic is. Fig 3 shows that the final size exhibits a sharp transition at a critical value of *ρ*_0_ in all the four cases with different combinations of the latent period (*τ*_E_ = 1,20) and the mobility reduction (α = 0,1). The epidemic threshold is larger for a shorter latent period and a larger mobility reduction rate. The significant increase in the epidemic threshold is observed only when the latent period is short (*τ*_E_ = 1) and the mobility reduction is full (α = 0). The short latent period limits the area where the exposed individuals move around, and as a result, the region where they turn to infectious individuals becomes small. On the other hand, the large mobility reduction prevents the infectious individuals from moving around in a wide region. If either one of the two conditions is not satisfied, the infectious individuals contact with many susceptible individuals, causing a large-scale outbreak. This result suggests that the social distancing of infectious individuals, such as travel restrictions and quarantine, is not so much effective for infectious diseases with a long latent period. We infer that the mobility of exposed individuals is also required to be reduced for diminishing the risk of an outbreak if the latent period is long.

### The characteristic length associated with the final epidemic size

To clarify how the final size depends on the mobility parameters, we consider the mobility of each individual as a random walk. We introduce the characteristic length *l* which represents the distance that the pathogens are carried by a single infected individual. The typical distance that an individual moves with a hopping rate *λ* during time *τ* is given by $2\sqrt{3\lambda \tau }$ (see Appendix for the derivation). Thus, we introduce a characteristic length *l* for the latent and infectious periods as follow:

$$l=2\sqrt{3\lambda (\tau_{E}+\alpha \tau_{I})},$$ (1)

because the hopping rate for the latent period is *λ* and that for the infectious period is *αλ*. During the infectious period, the frequency of infection events is influenced by the mobility of susceptible individuals who come to the site where infectious individuals are present. To evaluate the effective range that the pathogens can reach during the latent and infectious periods, we correct the characteristic length in Eq 1 as follows:

$$l^{*}=2\sqrt{3\lambda (\tau_{E}+(1+\alpha )\tau_{I})},$$ (2)

where the movement of susceptible individuals with hopping rate *λ* during the period *τ*_I_ is counted as an additional movement of infectious individuals with the same hopping rate in the same duration.

**Fig 4:**
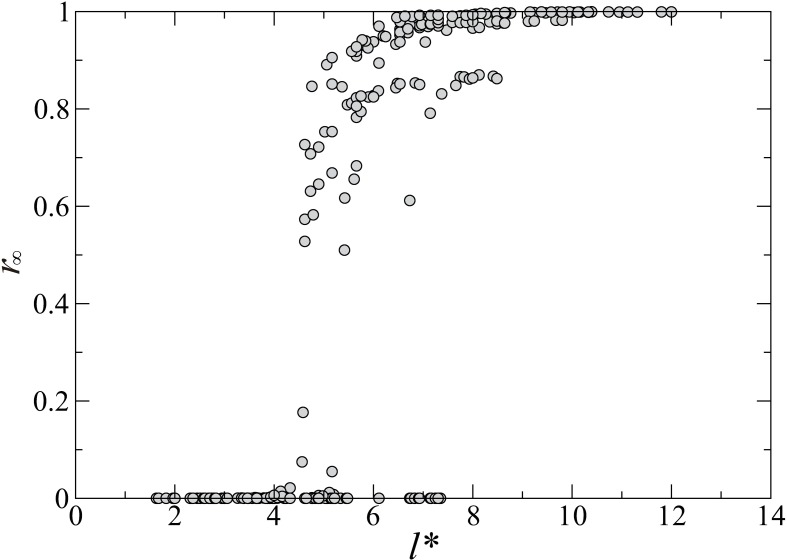
The dependence of the final size on the corrected characteristic length. The final size ***r***_∞_ computed with 375 parameter sets, including all possible combinations of ***τ***_**E**_ = **2,4,8,16,32**, $\tau_{I}=2,4,8,16,32,\;\lambda =\frac{1}{8},\frac{1}{9},\frac{1}{18}$, and ***α*** = **0,0.1,0.5,0.9,1**, are plotted against the corrected characteristic length l* given in Eq 2.

Now we numerically investigate the dependency of the final size on the corrected characteristic length l*. The computed final size *r*_∞_ is a randomly sampled value from 100 simulation results with different initial conditions for each set of parameter values. Fig 4 shows that *r*_∞_ depends strongly on the corrected characteristic length for the latent and the infectious periods. In this figure, the values of the final size are superimposed for 375 parameter sets including all possible combinations of *τ*_E_ = 2,4,8,16,32, $\tau_{I}=2,4,8,16,32,\;\lambda =\frac{1}{8},\frac{1}{9},\frac{1}{18}$, and *α* = 0,0.1,0.5,0.9,1. The result shows that all data points approximately collapse to a single curve. This means that the final size can be characterized by the single quantity l*.

**Fig 5:**
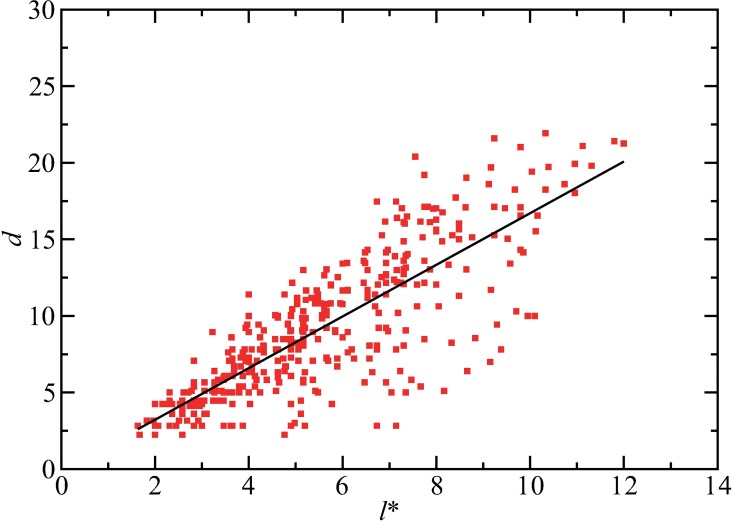
The correlation between the corrected characteristic length and the transport distance of the pathogens in the initial stage. The numerically computed values of the transport distance *d* is plotted against the corrected characteristic length l*. The parameter values are the same as those used in Fig 4. The straight line indicates the result of line fitting for the data, represented as *d* = 1.69l* - 0.15.

Next, the relationship between the corrected characteristic length and the infection process is examined. We introduce a distance *d* that represents the maximum distance between the initial position of the initial infectious individual and the position at which an individual who was infected by a contact with the initial infectious individual changes to an infectious individual. In other words, this distance measures the strength of the spatial diffusion of the pathogens carried by individuals that are infected initially. Fig 5 shows that *d* is positively correlated with the corrected characteristic length l*. The line fitting indicates approximately a linear correlation between l* and *d*. Therefore, it is validated that the characteristic length l* in Eq 2 plays an important role in the diffusion processes of the infection events, and thus, in the final size.

### An index associated with the final epidemic size

**Fig 6:**
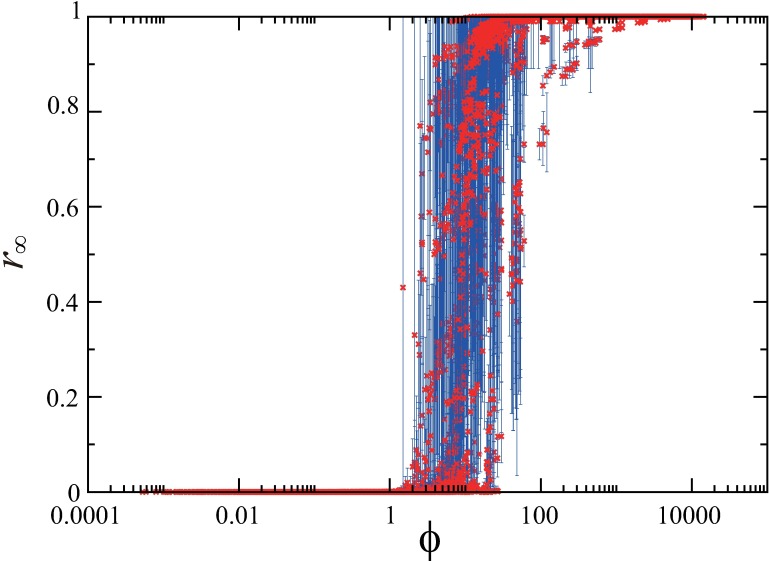
A scaling property for the final size. The average of the final size ***r***_∞_ over 100 trials with the error bar indicating the standard deviation is plotted against the index ***ϕ*** which is a function of the characteristic length l*, the transmission probability *p*, and the population density ***ρ*_0_**. The data points correspond to 12600 parameter sets, *L =* 100 and *n =* 10^3^, 10^4^, 10^5^, 10^6^, and *L* = 500 and *n =* 10^3^, 10^4^, 10^5^, for all the combination of ***τ*_E_ = 0,2,4,8,16,32, *τ*_I_ = 2,4,8,16,32**, *p =* 0.1, 0.5, 0.9, 1, $\lambda =\frac{1}{8},\frac{1}{9},\frac{1}{18}$, and ***α* = 0,0.1,0.5,0.9,1**.

In order to describe the essential contributions of the parameters more thoroughly by adding the effects of the population density *ρ*_0_ and the transmission probability *p* to the corrected characteristic length, we define an index *ϕ* as follows:

$$\phi \equiv {l^{*}}^{2}p\rho_{0},$$ (3)

which contains the contributions of all the parameters, *ρ*_0_, *τ*_E_, *τ*_I_, *p*, *λ*, and *α*. This index is defined based on the following considerations: (i) the final size would be proportional to the maximum size of the area where an exposed individual can move around before becoming an infectious one, i.e. *d*^*2*^ ~ l*^2^; (ii) the final size would be proportional to the transmission probability *p* as in the basic reproduction number that gives the epidemic threshold in the standard SEIR population model [38]; (iii) the final size would be proportional to the density *ρ*_0_ of the initial susceptible individuals. Fig 6 shows the relationship between *ϕ* and *r*_∞_, where the data points correspond to 12600 parameter sets, *L =* 100 and *n =* 10^3^, 10^4^, 10^5^, 10^6^, and *L* = 500 and *n =* 10^3^, 10^4^, 10^5^, for all the combination of *τ*_E_ = 0,2,4,8,16,32, *τ*_I_ = 2,4,8,16,32, *p =* 0.1, 0.5, 0.9, 1, $\lambda =\frac{1}{8},\frac{1}{9},\frac{1}{18}$, and *α* = 0,0.1,0.5,0.9,1. For each parameter set, the average of the final size *r*_∞_ over 100 trials is plotted with the error bar indicating the standard deviation. In spite of the great variability of *ϕ* over these parameter sets, the plotted points of the final size in Fig 6 show much less variability. For instance, the difference in the transition points of *ρ*_0_ for the four cases in Fig 3 is significantly reduced by the transformation in Eq 3: the numerically obtained critical values of *ϕ* are given by 17.9, 8.53, 9.84, and 10.56 for (*α*,*τ*_*E*_) = (0, 1), (0, 20), (1, 1), and (1, 20), respectively. The result indicates that the final size *r*_∞_ can be expressed approximately as a function of *ϕ*. The approximate invariance of the shape of the function under the scaling with *ϕ* implies a universal property of the epidemic spreading process.

Near the epidemic threshold, the fluctuation of the final epidemic sizes for the 100 trials tends to be large. The susceptibility measure and the variability measure are often used to characterize the sample-to-sample variability and numerically estimate the epidemic threshold [43–45]. Thus, we calculated these two measures with variation of the index *ϕ* as shown in Fig A of S1 File. The results show that the susceptibility measure well captures the large fluctuations near the epidemic threshold, corresponding to the state transition in Fig 6, although our index may be unsuitable for rigorous identification of the epidemic threshold.

So far we have assumed that the destination of the mobility is restricted to the neighbouring sites. However, in reality, there could be a more distant movement in a unit time. Thus, we have examined how our index in Eq 2 changes when the hopping to a more extended area is allowed. The results show that the index is qualitatively the same as Eq 2 but with different coefficients (see S1 File for the details).

### Correlation between the index and the incidence

Here we analyse a correlation between the index *ϕ* and the incidence of infectious diseases using real data. We focus on influenza B as one of the infectious diseases which cause outbreaks all over the world and are not responsible for recent pandemics. We suppose that an outbreak in the spatial SEIR model as found in Fig 2(E) is repeated every year for influenza B in the real world. For estimating *ϕ* from real data, we employ human mobility data through international airline networks.

We extracted the data of the number of specimens positive for influenza B from 2000 to 2014 in 44 countries from WHO statistics (FluNet) [46], after eliminating the countries for which the data in some years within the 15 years are unavailable or missing. The number of positive cases is deeply related to the incidence rate of influenza B. We denote the total number of positive cases in year *Y* by *N*_C_(*Y*) for *Y* = 2000, …, 2014. Since in some countries the outbreak of influenza B shows biennial patterns caused by outbreak periods including the year end, we smoothed the total number of positive cases by averaging those in the previous, current, and next years as follows: ${\bar{N}}_{C}(Y)=(N_{C}(Y-1)+N_{C}(Y)+N_{C}(Y+1))/3$ for *Y* = 2001, …, 2013. The time series of the smoothed data shows that the number of positive cases of influenza B has an upward trend, particularly in recent several years.

To examine the trend of influenza B incidence from the proposed index *ϕ*, we extracted the data of the number of international airline passengers [47] and the total population in 214 countries [48] from the World Bank, after eliminating the airlines for which the data in some years are missing. We denote the number of the international airline passengers and the total population in year *Y* by *N*_P_(*Y*) and *N*(*Y*), respectively, for *Y* = 2001, …, 2013. The population density is approximated as *ρ*_0_∼*N*(*Y*)/*R* where *R* is the size of the habitation area. The hopping rate, approximately corresponding to the probability that the individuals move to other places by flights, is given by λ∼*N*_P_(*Y*)/*N*(*Y*). Therefore, it follows that *ρ*_0_λ ∝ *N*_P_(*Y*). From Eq 3, we obtain the proposed index for year *Y* as follows:

$$\phi (Y)\propto \phi_{e}(Y)\equiv N_{P}(Y)(\tau_{E}+(1+\alpha )\tau_{I}).$$ (4)

**Fig 7:**
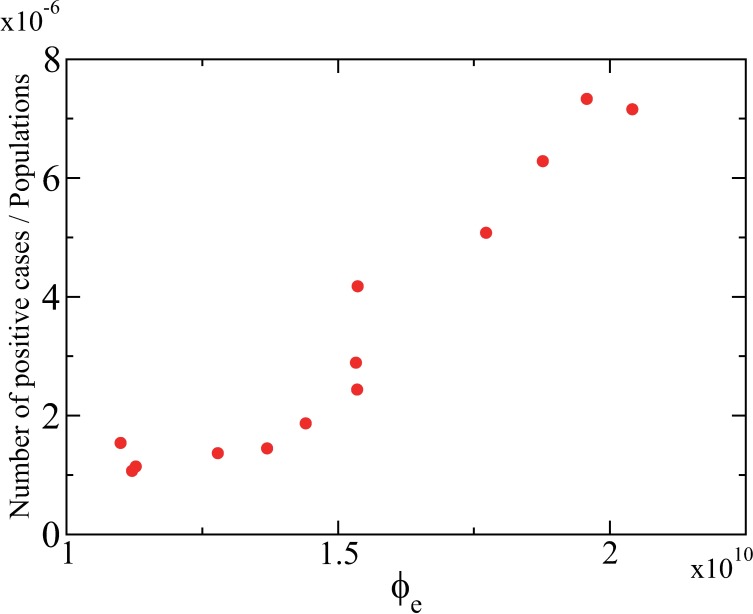
Correlation between the proposed index and the positive incidence of influenza B. The number of positive cases of influenza B was obtained from the FluNet database [46] and was divided by the total population [48] to eliminate the influence of the population increase. The proposed index was estimated by using the data of the number of passengers in international airlines [47] and the total population. A strong positive correlation between the proposed index and the incidence of influenza B implies that the increased human mobility is responsible for the growing number of the incidence.

Assuming that the number of positive cases is proportional to the final size multiplied by the total population and the final size tends to increase with the index *ϕ*, we roughly assume that the number of positive cases is represented as follows:

$${\tilde{N}}_{C}(Y)=A{N(Y)N}_{P}(Y)(\tau_{E}+(1+\alpha )\tau_{I}),$$ (5)

where *A* is a scaling parameter. We assume that the latent and infectious periods are set at *τ*_E_ = 2 (days) and *τ*_I_ = 4 (days) [49]. Then, we numerically fitted the unknown parameters *A* and α using the gradient-based method so that the total error

$$\sum_{Y=2001}^{2013}{\left|{\tilde{N}}_{C}(Y)-{\bar{N}}_{C}(Y)\right|}^{2}$$ (6)

is minimized. Using the estimated parameter values of *A* and *α*, we can obtain the value of *ϕ*_*e*_ defined in Eq 4. Fig 7 shows the positive correlation between the estimated value of *ϕ*_*e*_ and the incidence. The incidence rate of positive cases was calculated from the smoothed number ${\bar{N}}_{C}$ of cases of influenza B by normalizing with the total population in the world. By this operation, we can eliminate the influence of the population growth. The result suggests that the recent increase in the incidence of influenza B attributes to the increased frequency of human travels. The index *ϕ* including both the disease-dependent property and the human mobility effect is useful for estimating the trend of the incidence of infectious diseases in modern societies with globalized human mobility.

## Discussion

We have investigated the spatial SEIR model with stochastic mobility of individuals on the square lattice to reveal how heterogeneous spatial mobility influences the final epidemic size. Our model is characterized by six parameters: the initial density of susceptible individuals, the length of latent period, the length of infectious period, the transmission probability, the hopping rate, and the mobility reduction rate. The main contribution of this study is the proposal of the index *ϕ* as an explicit function of these six parameters, which is largely associated with the final epidemic size. Through this index, it is obvious how parameter scaling changes the final epidemic size. This index can be regarded as a control parameter governing the final epidemic size in the presence of spatial mobility of individuals.

It has been commonly recognized that the factors promoting the spreading of epidemics include the transmission rate and the population density [4, 5] in many types of epidemic models, but the role of human mobility is still not fully elucidated. In this study, we have illustrated that the mobility parameter interacts with the latent period and therefore the mobility reduction of infectious individuals is effective only in the case with a short latent period. This result implies the limitation of social distancing measures including travel restrictions and quarantine. For infectious diseases with a long latent period, a countermeasure to reduce the mobility of both exposed and infectious individuals would be beneficial.

The proposed index is based on the idea that the final size is largely governed by the effective transport distance that the pathogens can reach during the latent and infectious periods. The transport distance is well correlated with the characteristic length of the human mobility, which is theoretically derived using the analysis of diffusion equations. Therefore, the proposed index is represented using the characteristic length and the other system parameters. In the proposed index, the interplay between the mobility parameter and the other epidemiological parameters is clear.

We have demonstrated that the index *ϕ* incorporating the mobility effects is strongly correlated with the actual number of the incidence of past influenza epidemics using the mobility data through airline travels. This result indicates a possibility that the increasing trend of the influenza incidence in recent years is attributed mainly to the expansion of the human travels.

Our spatial epidemic model explicitly considers the spatial movement of individuals, and therefore, belongs to the class of individual-based models. Instead of conducting a realistic simulation with the individual-based model as in many previous studies, we have explored a universal property with regard to the final epidemic size by assuming the simple hopping rule from site to site. In this study, the key index *ϕ* has been introduced based on the numerical observations. One of the future issues is to derive a similar index based on analytical calculations and compare the index with those for other models.

The proposed model can be regarded as a generalization of some existing models, because it is reduced to a well-mixed population model if the number of sites is set at one and also equivalent to a spatial metapopulation model if each site contains a sufficiently large subpopulation. So far spatial metapopulation models have been mainly employed for realistic simulations with mobility data, but theoretical analyses of them have received less attention. Our approach in this study, which has led to parameter scaling for the final epidemic size resulting from an interaction between epidemiological and mobility parameters, could be a first step to develop a more general and theoretical understanding of epidemic spreading in spatial metapopulation models with heterogeneous human mobility [50]. A possible direction is to construct a spatial epidemic model to which the mathematical theory of spatial diffusion processes is applicable, in order to analyse the effect of heterogeneous mobility which largely influences the final epidemic size.

## Appendix

We show that the characteristic length which a random walker with hopping rate *λ* reaches in time interval *τ* in a two-dimensional lattice is approximately given by $2\sqrt{3\lambda \tau }$ in the following calculations.

Let *C*(*x*,*y*,t) be a probability density function (PDF) of random walkers in a two-dimensional space with coordinate (*x*, *y*). The diffusion equation for the PDF is written as follows:

$$\frac{\partial C}{\partial t}=D\left(\frac{\partial^{2}C}{\partial x^{2}}+\frac{\partial^{2}C}{\partial y^{2}}\right),$$

where *D* denotes the diffusion constant. When the initial condition is given by *C*(*x*,*y*,0) = δ(*x*,*y*) where δ is the Dirac’s delta function, we can solve the diffusion equation for *C*(*x*,*y*,t) using the Fourier transform and obtain the solution as follows:

$$C(x,y,t)=\frac{1}{4\pi Dt}e^{-\frac{\left(x^{2}+y^{2}\right)}{4Dt}},$$

which is called a normalized Gaussian function [51]. This solution can be rewritten using the polar representation with *x* = *r* cos *θ* and *y* = *r* sin *θ* as follows:

$$C(r,\theta ,t)=\frac{1}{4\pi Dt}e^{-\frac{r^{2}}{4Dt}}.$$

Therefore, the characteristic length for time interval *τ* in the PDF is given by $2\sqrt{D\tau }$.

If we have a parameter which corresponds to *D* in our two-dimensional lattice model, its characteristic length can be represented with the parameter. By discretizing the space and time with *t* ≡ *n*Δ*t*, *x* ≡ *i*Δ*x*, and *y* ≡ *j*Δ*x*, the time evolution of the PDF of random walkers, *u*_*i*,*j*_(*t*), in the two-dimensional lattice can be represented as follows:

$$u_{i,j}(t+∆t)-u_{i,j}(t)=-8\lambda u_{i,j}(t)+\lambda \left(u_{i+1,j}(t)+u_{i-1,j}(t)+u_{i,j+1}(t)+u_{i,j-1}(t)+u_{i+1,j+1}(t)+u_{i+1,j-1}(t)+u_{i-1,j+1}(t)+u_{i-1,j-1}(t)\right).$$

The first term in the righthand side represents the outflow from the site (*i*, *j*) and the other terms represent the inflow from the eight neighbouring sites. By neglecting high-order terms after the Taylor expansion, the previous equation is approximated as follows:

$$\frac{\partial u(x,y,t)}{\partial t}≅\frac{3\lambda {\left(\Delta x\right)}^{2}}{\Delta t}\left(\frac{\partial^{2}u(x,y,t)}{\partial x^{2}}+\frac{\partial^{2}u(x,y,t)}{\partial y^{2}}\right).$$

Comparing this with the original diffusion equation, we obtain $D≅\frac{3\lambda {\left(\Delta x\right)}^{2}}{\Delta t}$. When the unit time is set at Δ*t* = 1 and the unit length is at Δ*x* = 1, it follows *D* ≅ 3*λ*. Hence, the characteristic length of the random walk for time interval *τ* in the two-dimensional lattice is approximately given by $2\sqrt{3\lambda \tau }$.

## Supporting Information

S1 File The supporting information file includes Figs A-D, in addition to some additional explanations. (PDF)
