# Supplementary material for: Parameter Scaling for Epidemic Size in a Spatial Epidemic Model with Mobile Individuals
Source: PLoS One. 2016 Dec 14;11(12):e0168127. doi: 10.1371/journal.pone.0168127 (PMC5156435; doi:10.1371/journal.pone.0168127)
Supplement: S1 File — (PDF) [file pone.0168127.s001.pdf]

Supplementary Information for "Parameter Scaling for Epidemic Size in a Spatial Epidemic Model with Mobile Individuals"

Chiyori T. Urabe[1*], Gouhei Tanaka[1,2], Kazuyuki Aihara[1,2], Masayasu Mimura[3]

[1] Institute of Industrial Science, The University of Tokyo, Tokyo 153-8505, Japan

[2] Graduate School of Engineering, The University of Tokyo, Tokyo 113-8656, Japan

[3] Meiji Institute for Advanced Study of Mathematical Sciences, Meiji University, Tokyo 164-8525, Japan

List of contents

1. Variability of the final epidemic size near the epidemic threshold
2. Mobility with hopping to an extended area

1. Variability of the final epidemic size near the epidemic threshold

To characterize the sample-to-sample variability near the epidemic threshold, we computed the susceptibility measure defined as $\chi = N(\langle r_\infty^2\rangle - \langle r_\infty\rangle^2)/\langle r_\infty\rangle$ and the variance measure defined as $\Delta = \sqrt{\langle r_\infty^2\rangle - \langle r_\infty\rangle^2}/\langle r_\infty\rangle$, where N is the system size, r_∞ represents the final epidemic size, and the brackets indicate the sample average over the 100 trials.

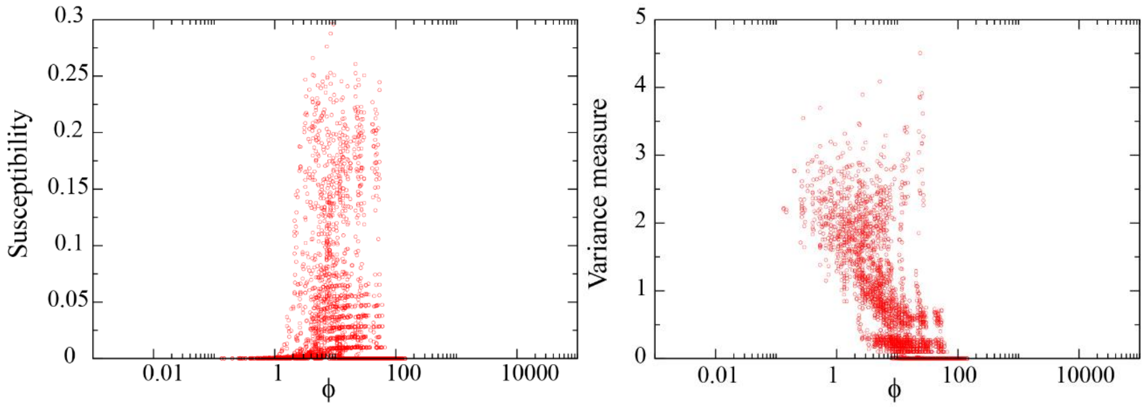

Figure A. The susceptibility measure (Left) and the variability measure (Right) calculated with the outbreak size under the variation of the index ϕ. The parameter sets are all the combinations of $L = 100$, $n = 10^5$, $\alpha = 0, 0.1, 0.5, 0.9, 1$, $\lambda = \frac{1}{8}, \frac{1}{9}, \frac{1}{18}$, $\tau_E = 0, 2, 4, 8, 16, 32$, and $\tau_I = 2, 4, 8, 16, 32$.

For the susceptibility measure, we set $N = 1$ for simplicity because the system size is fixed at $L = 100$ and $n = 10^5$. These two measures are plotted against the proposed index ϕ as shown in Fig A. The results show that the range of ϕ (between approximately 1 and 100) for large values of the susceptibility measure (Fig A, Left) is in better agreement with that for the large standard deviation of the final size in Fig 6 than that for the large values of the variance measure (Fig A, Right). Therefore, it is possible that the epidemic threshold exists within the range of ϕ and the susceptibility measure captures the large fluctuations near the threshold.

2. Mobility with hopping to an extended area

2.1 Model

In the main text, the destination of the hopping of each individual is limited to the 8 sites surrounding the current site. However, in a more realistic case, the hopping to more distant sites would be possible. To investigate how our results change in such a case, here we consider the hopping to an extended area.

As illustrated in Fig B, the destination of the hopping includes the 16 more distant sites (dashed arrows) in addition to the original 8 sites (solid arrows). When susceptible, exposed, and recovered individuals are located in a site (i, j), each individual can hop to one of the 8 sites at $(\mathrm{i} \pm 1, \mathrm{j} \pm 1), (\mathrm{i} \pm 1, \mathrm{j})$, and $(\mathrm{i}, \mathrm{j} \pm 1)$, with hopping rate λ or one of the 16 sites at $(\mathrm{i} \pm 2, \mathrm{j} \pm 2), (\mathrm{i} \pm 2, \mathrm{j} \pm 1)$, $(\mathrm{i} \pm 2, \mathrm{j}), (\mathrm{i} \pm 1, \mathrm{j} \pm 2)$, and $(\mathrm{i}, \mathrm{j} \pm 2)$ with hopping rate $\lambda/2$. For infectious individuals, the hopping rates are multiplied by α, where $1 - \alpha$ represents the mobility reduction rate.

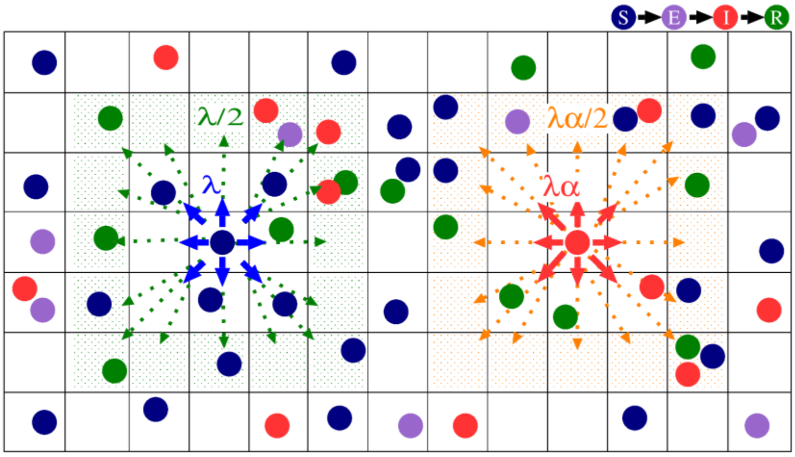

Figure B. Schematic illustration of the spatial SEIR model with hopping to an extended area in the square lattice. Each individual randomly hops from site to site. Each of the susceptible (S), exposed (E), and recovered (R) individuals hops to one of the 8 sites indicated by solid arrows with hopping rate λ or one of the 16 sites indicated by

dashed arrows with rate $\lambda/2$. For infectious individuals, the hopping rate is multiplied by the factor α where $1-\alpha$ represents the mobility reduction rate.

2.2 Correlation between the characteristic length and the transport distance

For the hopping to the extended area, the typical distance that an individual moves with a hopping rate λ during time τ is given by $2\sqrt{14\lambda\tau}$ which is derived in the same manner as in Appendix in the main text. Therefore, the characteristic length l_{ext}^{*}, which represents the effective range that the pathogens are transported during the latent and infectious periods, τ_E and τ_I, is given as follows:

$$l_{\text{ext}}^{*} = 2\sqrt{14\lambda(\tau_E + (1+\alpha)\tau_I)}. \tag{S1}$$

We can confirm the positive correlation between l_{ext}^{*} and the transport distance d (see the main text for the definition) as shown in Fig C, as in the case of local hopping (Fig 5). This means that the transport distance that pathogens are carried by infection increases with the characteristic length l_{ext}^{*}.

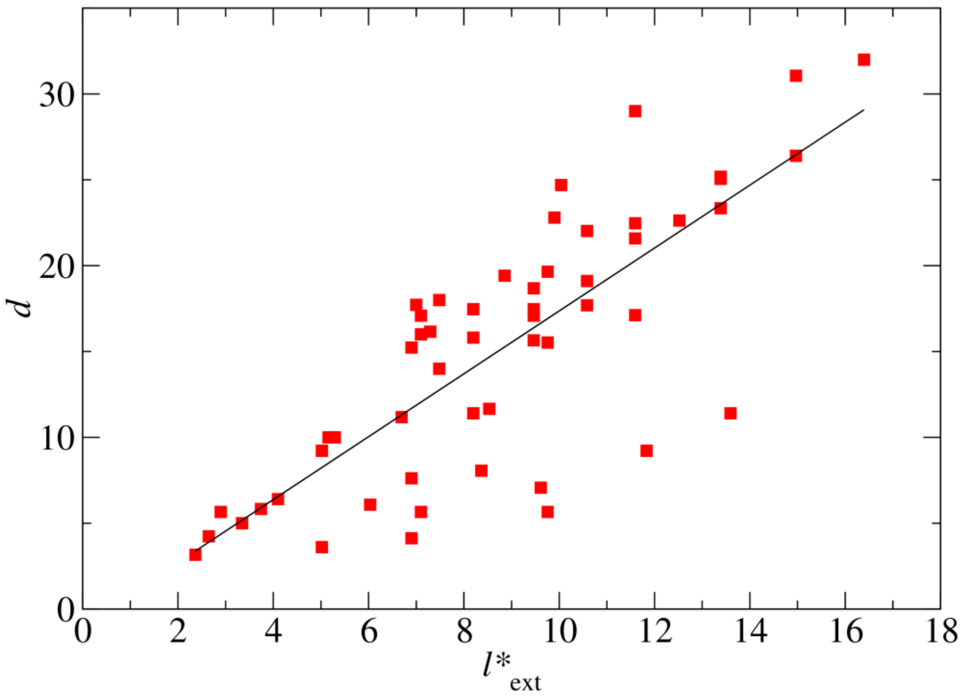

Figure C. The correlation between the characteristic length l_{ext}^{*} and the transport distance d of the pathogens in the initial stage. The parameter values are all the combinations of $L = 500, n = 10^5, p = 1, \alpha = 0, 0.5, 1, \lambda = 0.025, 0.05, \tau_E = 2, 16, 32$, and $\tau_I = 2, 16, 32$. The straight line indicates the result of line fitting for the data, represented as $d = 1.83 l_{\text{ext}}^{*} - 0.949$.

2.3 Relationship between the index ϕ_{ext} and the final size r_∞

In the main text, the final size of epidemics r_∞ is approximated by a function of the index ϕ as shown in Fig 6. In the cases with hopping to the extended area, the index can be rewritten using the characteristic length l^*_{ext} as follow:

$$\phi_{\text{ext}} \equiv {l^*_{\text{ext}}}^2 p\rho_0. \tag{S2}$$

The relationship between the index ϕ_{ext} and the final size r_∞ is as shown in Fig D. The shape of a function in Fig D is similar to Fig 6. The more index increases, the more the epidemic tend to spread, while the threshold is different from that in Fig 6.

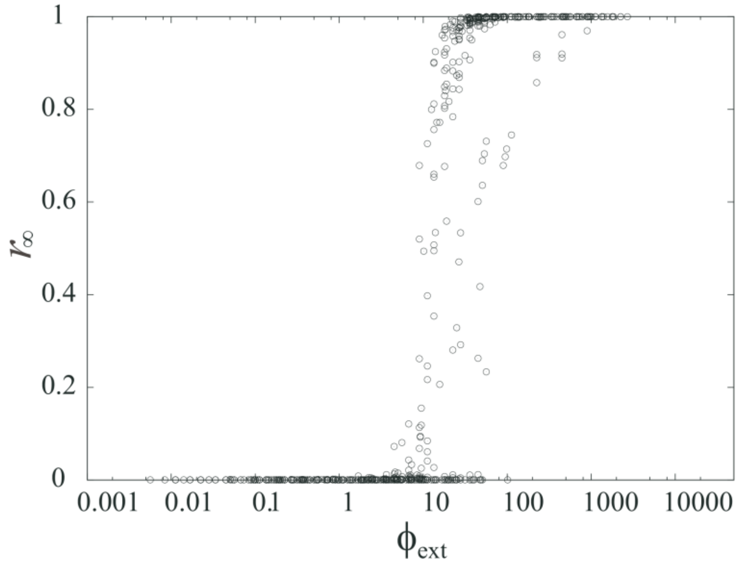

Figure D. A scaling property for the final size. The numerically computed values of the maximum final size r_∞ are plotted against the index ϕ_{ext}. The parameter sets are given by all the combinations of $L = 100, 500,$ $n = 10^3,\ 10^4,\ 10^5,$ $\tau_E = 0, 2, 16, 32,$ $\tau_I = 2, 16, 32,$ $p = 0.5,$ $\lambda = 0.025, 0.05,$ and $\alpha = 0, 0.5, 1.$

2.4 Discussion

As an example of extended mobility, we have considered the cases of hopping to the extended area. The maximum distance that each individual can move at a unit time is doubled, compared with that for the hopping to neighbouring sites as studied in the main text. We have derived the characteristic length and shown that the transport distance that pathogens are carried by an infected individual is proportional to the characteristic length. Therefore, the index associated with the final epidemic size can be similarly formulated as in the case of the main text, which can predict the effect of parameter scaling on the final size. It is an interesting future issue to consider an index for other types of spatial mobility.
